# tRNA-derived small RNAs in ocular neovascular diseases: A systematic review

**DOI:** 10.1016/j.ncrna.2026.03.004

**Published:** 2026-05-23

**Authors:** Tian-yi Liu, Peng-zhou Kuai, Yi-sheng Luo, Yu Song, Yong Wang, Xiao-bo Huang, Xin Cao

**Affiliations:** a Department of Ophthalmology, The Second Affiliated Hospital of Nantong University and First People's Hospital of Nantong City, Nantong, Jiangsu, China; b Department of Ophthalmology, Southeast University Affiliated Nantong First People's Hospital, Nantong, Jiangsu, China

**Keywords:** Ocular neovascular diseases, tsRNA, Epigenetics, Angiogenesis

## Abstract

Ocular neovascular diseases (ONDs) are a leading cause of global blindness. While current anti-vascular endothelial growth factor (VEGF) treatments have revolutionized management, their efficacy is constrained by issues such as non-response, resistance development, and the burden of repeated intravitreal injections. Recent studies have highlighted the growing importance of transfer RNA-derived small RNAs (tsRNAs) in regulating pathological angiogenesis. This review provides a systematic analysis of tsRNAs, encompassing their origins, classification, and mechanisms of action, followed by an exploration of their implications in OND. Targeting tsRNAs may offer a novel multi-level regulatory strategy to overcome the limitations of current anti-VEGF therapies.

## 1 Introduction

OND represent a group of blinding eye conditions characterized by the pathological formation of new blood vessels originating from pre-existing abnormal vasculature. OND primarily includes diseases such as diabetic retinopathy (DR), neovascular age-related macular degeneration (NV-AMD) and corneal neovascularization (CoNV). OND often arises secondary to diabetes, inflammation, hypoxia, or other pathological conditions, posing a substantial burden to global eye health [1]. The advent of anti-VEGF agents has revolutionized the therapeutic landscape for intraocular neovascular diseases [2]. In recent years, novel therapeutics targeting alternative pro-angiogenic pathways—such as angiopoietin (ANG) and platelet-derived growth factor (PDGF)—have been under active investigation. These emerging strategies offer potential benefits for patients with suboptimal response to anti-VEGF therapy and may mitigate adverse effects associated with its long-term administration [3].

The development of RNA sequencing technologies has enabled the identification of highly evolutionarily conserved small non-coding RNAs (sncRNAs), including tsRNAs or tRNA-derived fragments (tRFs), across various species [4]. tsRNAs are produced through the cleavage of precursor or mature tRNAs at specific sites by dedicated enzymes and play important roles in biological regulation. They have been closely linked to multiple diseases [5], including cancer [6], neurodegenerative disorders [7], and stress-induced injury [8]. Their mechanisms of action encompass gene silencing [9], modulation of protein translation [10,11], and competitive binding to essential proteins [12], among others. As a result, tsRNAs show promise as novel biomarkers and therapeutic targets. In recent years, growing efforts have been made to extend tsRNA research into the field of ophthalmology, particularly in neovascular diseases [13].

To identify publications aligned with our focus, a systematic search was performed in databases including PubMed with keywords pertaining to tsRNAs and retinal vascular disorders. Each publication was carefully evaluated to confirm its direct relevance, and those meeting the inclusion criteria—specifically investigating the function of tsRNAs in the context of ocular neovascularization—were selected for further analysis. We examined how alterations in tsRNAs affect angiogenesis and explored the underlying molecular mechanisms involved. Furthermore, we summarized recent advances concerning the role of tsRNAs in the diagnosis and treatment of OND, thereby contributing to a deeper understanding of these diseases and suggesting novel therapeutic directions.

## 2 Biogenesis and regulatory mechanisms of tsRNA

### 2.1 Classification and biogenesis pathways of tsRNA

**Fig. 1:**
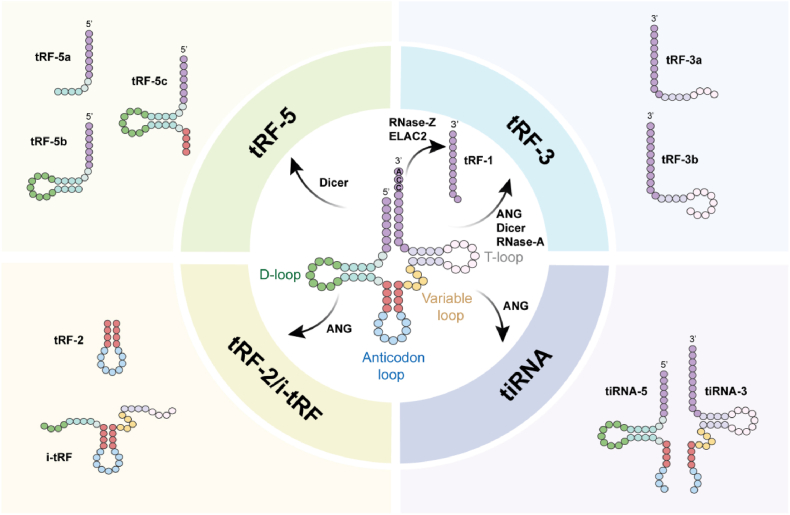
Classification of tsRNA. tsRNAs are produced through enzymatic cleavage of precursor or mature tRNAs under basal and stressed conditions, and fall into two primary categories: tRNA halves, which are stress-induced and include 3′- and 5′- tiRNAs, and tRFs, which are systematically classified by cleavage site into tRF-5, tRF-3, and tRF-1. This classification also encompasses atypical variants such as internal tRFs (i-tRFs).

tsRNAs represent a group of highly conserved non-coding RNAs generated by the enzymatic cleavage of precursor or mature tRNAs under both normal and stressed conditions. Based on structural characteristics and biogenetic mechanisms, tsRNAs are mainly divided into two broad types: tRNA halves and tRFs [14]. A detailed classification tree of tsRNAs, including tRNA halves and tRFs along with their respective subclasses, is presented in Fig. 1.

A body of evidence indicates that tRNA halves are present at low abundance in human cell lines under basal conditions, but are significantly upregulated in response to oxidative stress, nutrient deprivation, hypoxia, heat shock, and other stressors. ANG cleaves mature tRNAs in the anticodon loop, generating tRNA halves approximately 30–40 nt in length, suggesting their important role in stress-related diseases and tissue injury. Consequently, these tRNA halves are also referred to as tRNA-derived stress-induced RNAs (tiRNAs) [15]. tiRNAs are further classified into two subclasses: 3′-tiRNAs and 5′-tiRNAs, which are derived from opposing ends of the tRNA. 3′-tiRNAs originate in the anticodon loop and extend toward the 3′ terminus, while 5′-tiRNAs initiate at the 5′ end and conclude within the anticodon region [16]. The expression levels of tRNA halves vary considerably across conditions, tissues, species, and depending on their 5′ or 3′ origin, and do not always correlate with the expression of their corresponding mature tRNAs, underscoring the specificity of their biogenesis [17].

The other class of tRFs are 14-30 nucleotides long and comparable in size to miRNAs [18]. Based on cleavage sites, tRFs are systematically classified into three major types: tRF-5, tRF-3, and tRF-1. tRF-5 fragments are derived from the 5′ end of tRNAs through enzymatic processing within the D-loop or the adjacent stem region near the anticodon loop, and are further differentiated into subtypes tRF-5a, 5b, and 5c according to specific cleavage loci. tRF-3 fragments are produced by endonucleases such as ANG, Dicer, or other RNase A superfamily enzymes via cleavage within the T-loop. Variations among tRF-3 species—including tRF-3a and tRF-3b—arise from differences in the precise cleavage site within the TΨC loop. tRF-1 fragments are generally several orders of magnitude less abundant than major tRF subtypes and are released during tRNA 3′ end maturation via cleavage by RNase Z/ELAC2 [19]. In addition to these canonical tRFs, there exists a class of low-abundance atypical tRFs (e.g., tRF-2) that can be detected through high-throughput sequencing, though the detailed ribonuclease mechanisms responsible for their biogenesis remain unclear [20]. Furthermore, internal tRFs (i-tRFs) represent a newly identified subclass derived from internal regions of mature tRNAs, whose length and expression levels have been closely linked to disease states [6].

### 2.2 Epigenetic regulation of tsRNA biogenesis by tRNA modifications

Post-transcriptional modifications of tRNAs serve as critical "molecular switches" in regulating tsRNA biogenesis, with methylation and demethylation processes playing particularly important roles. Methylation "writers" (e.g., methyltransferase families) and "erasers" (e.g., demethylases) modulate tRNA conformation through reversible modifications, thereby modulating their accessibility to nucleases [21]. These modifications generally enhance tRNA resistance to endonucleolytic cleavage and prevent its fragmentation. Specific modifications protect tRNAs from ANG-mediated cleavage by altering tRNA conformation or directly blocking endonuclease access, thereby reducing tsRNA production. Conversely, certain modifications can promote tsRNA biogenesis, collectively forming a sophisticated regulatory network [22].

Multiple tRNA modifications protect tRNAs from endonucleolytic cleavage by angiogenin (ANG) through enhancing tRNA stability or directly blocking cleavage sites, thereby reducing tsRNA production [23]. DNMT2-and NSUN2-mediated m^5^C modifications increase tRNA stability, and their deficiency renders tRNAs more susceptible to cleavage into tsRNAs [24]. QTRT1-catalyzed queuosine (Q) modification occurs at the anticodon wobble position of several tRNAs, protecting them from ANG-mediated cleavage [25], and Q modification has been shown to promote DNMT2-mediated m^5^C modification on the same tRNA [26]. Furthermore, elevated m^1^A levels resulting from ALKBH1/3 deficiency [27,28], TRMT10A-mediated m^1^G modification [29], and 2′-O-methylation at C34 of tRNA^Met^ all suppress tsRNA generation [30].

In contrast to the protective modifications described above, certain modifications can promote tRNA cleavage to enhance tsRNA production. Under oxidative stress conditions, downregulation of TRMT2A in mammalian cells induces m^5^U54 hypomodification, leading to ANG overexpression and subsequent accumulation of 5′tiRNAs [31]. PUS7-mediated pseudouridine (Ψ) modification at position U8 promotes the generation of specific 5′-tsRNAs in stem cells, and Pus7 deletion results in reduced levels of these tsRNAs [32]. In yeast, mcm^5^S^2^ modification at the anticodon wobble position similarly promotes tRNA cleavage [33]. Notably, tRNA cleavage can be reversible: after bacterial tRNAs are cleaved by ribotoxins, the Pnkp/Hen1 complex can rejoin tsRNA halves into intact tRNAs while adding a 2′-O-methylation at the cleavage site to prevent re-cleavage [34], revealing the dynamic reversibility of tsRNA biogenesis.

tRNA modifications constitute a sophisticated regulatory network that dynamically balances tRNA stability and tsRNA generation through the coordinated action of methyltransferases and demethylases. This network not only maintains cellular homeostasis but also mediates adaptive responses to environmental stressors, playing critical roles in development, metabolism, and disease pathogenesis. Modification status not only influences tsRNA generation but also determines the functional properties of the resulting tsRNAs. Moreover, tsRNAs may acquire additional modifications after being cleaved from precursor tRNAs, further increasing regulatory complexity. These RNA modifications pose technical challenges for tsRNA detection and functional studies, and addressing these challenges will be crucial for advancing the field.

### 2.3 Methodological advances in tsRNA profiling

A key recent trend in tsRNA research has focused on methodological improvements that overcome the blocking effects of internal and terminal RNA modifications, thereby reducing bias and enabling the detection of highly modified tsRNAs that were previously undetectable by conventional small RNA sequencing methods [35]. Conventional small RNA-seq requires 5′-monophosphate and 3′-hydroxyl termini for adapter ligation and is impaired by internal methylations that block reverse transcription. However, tsRNAs frequently harbor terminal modifications (e.g., 3′-phosphate, 2′,3′-cyclic phosphate) and internal methylations (m^1^A, m^3^C, m^1^G) that render them invisible to standard approaches [36].

Advanced techniques such as PANDORA-seq and CPA-seq overcome these limitations through combinatorial enzymatic treatments. PANDORA-seq employs T4 polynucleotide kinase to convert modified termini to ligation-competent ends and AlkB demethylases to remove reverse transcription-blocking methylations, enabling comprehensive detection of highly modified tsRNAs [35]. CPA-seq incorporates additional steps including deacylation and decapping to further expand detectable small RNA species [36].

The application of these improved methodologies has accelerated both mechanistic discovery and biomarker development. PANDORA-seq identified tsRNA-Glu-CTC as a cholesterol-responsive regulator of hepatic lipid metabolism and atherosclerosis in mice, demonstrating that endogenous RNA modifications confer enhanced bioactivity [37]. In reproductive biology, PANDORA-seq revealed an "aging cliff" in sperm tsRNA/rsRNA profiles during the 50–70 week transition and uncovered an evolutionarily conserved age-dependent length shift in rsRNAs. Notably, tsRNA/rsRNA cocktails mimicking aged sperm RNA profiles induced transcriptomic changes in metabolic and neurodegenerative disease pathways in embryonic stem cells, mirroring phenotypes observed in offspring fathered by aged sperm [38]. In cancer diagnostics, PANDORA-seq profiling of plasma extracellular vesicle-derived small RNAs established a three-rs/tsRNA signature that distinguishes gastric cancer patients from healthy controls with high sensitivity and specificity, including for early-stage disease [39]. Similar approaches have identified circulating tsRNA biomarkers in other malignancies [40], underscoring the translational potential of these technologies.

By overcoming the barriers imposed by RNA modifications, PANDORA-seq and related methods have fundamentally reshaped our understanding of the small RNA landscape and are poised to accelerate tsRNA-based biomarker and therapeutic development across human diseases, including ocular neovascular disorders.

## 3 Biological functions of tsRNAs

tsRNAs exhibit diverse biological functions by participating in transcriptional regulation, post-transcriptional modulation, and translation processes, thereby playing critical roles in gene expression control [41]. In addition to regulating gene expression, they participate in various other biological processes including immune response, apoptosis, embryonic transcriptional cascades, and cellular stress responses [42]. Aberrant tsRNA expression has been confirmed to correlate strongly with the pathogenesis of multiple diseases, underscoring their promise as biomarkers and therapeutic targets [43,44].

### 3.1 Transcriptional regulation

tsRNAs modulate transcriptional activity through effects on DNA methylation, histone modification, and non-coding RNA interactions, consequently orchestrating key cellular processes [45]. For example, in non-small cell lung cancer, AS-tDR-007333 interacts with HSPB1 and promotes MED29 expression by increasing H3K4me1 and H3K27ac modifications in the MED29 promoter region. It also upregulates the transcription factor ELK4, facilitating its binding to the MED promoter and enhancing transcriptional activation [46]. In another study, the tRNA-Glu-derived fragment td-piR(Glu) forms a complex with PIWI-like protein 4 and recruits SETDB1, SUV39H1, and heterochromatin protein 1β to the CD1A promoter region. This process enhances H3K9 methylation, leading to substantial transcriptional repression of CD1A and subsequent modulation of membrane protein expression [47]. Additionally, tsRNAs may bind to specific genomic DNA regions via base complementarity, interfering with transcription factor binding or RNA polymerase activity. As an example, tsRNA-GlyGCC enhances the advancement of colorectal cancer and confers resistance to 5-fluorouracil chemotherapy through modulation of the oncogenic transcription factor SPIB [48].

### 3.2 Post-transcriptional regulation

tRFs modulate post-transcriptional gene expression through interactions with RNA-binding proteins. Specifically, by associating with Argonaute (AGO) proteins and assembling into the RNA-induced silencing complex (RISC), they facilitate mRNA targeting via complementary base pairing with 3′ untranslated regions (3′ UTRs), resulting in suppression of gene expression [49]. For example, tRF-3017A inhibits NELL2 expression in gastric cancer through its incorporation into the RISC complex alongside AGO proteins, consequently restraining tumor migration and invasive capacity [50]. Beyond the Argonaute family, tRFs are capable of engaging with a diverse array of RNA-binding proteins (RBPs). Certain tRFs and tiRNAs enhance cellular proliferation and drive cell cycle advancement through association with RNA-binding proteins like Y-box binding protein 1 (YBX1), resulting in transcriptional inhibition [51]. Additionally, tiRNA-Val-CAC-2 specifically interacts with the KH3-KH4 domains of FUBP1, thereby stabilizing the protein and promoting its accumulation at the c-MYC promoter. This interaction ultimately activates c-MYC transcription and drives metastasis in pancreatic cancer [52].

### 3.3 Translational regulation

tsRNAs modulate mRNA translation efficiency by binding to ribosomes or competitively inhibiting tRNA function. They are capable of disassembling the translation initiation complex from the 5′ cap structure of mRNAs, thereby globally suppressing protein synthesis under stress conditions to promote cell survival [53]. For example, the 3′tsRNA derived from tRNALeuCAG enhances the translation of RPS28—a protein essential for ribosomal subunits—thereby augmenting overall protein synthesis in vertebrates [54]. Another study found that 3′-tiRNAThr facilitates mRNA loading onto ribosomes by interacting with ribosomal complexes, thus promoting translation [55]. Beyond modulating ongoing translation, tsRNAs can also interfere with translation initiation. For instance, 5′-tiRNA-Gln binds to eIF4A1, an RNA helicase required for initiation, inhibiting the process and reducing the production of proteins associated with hepatocellular carcinoma progression [56].

### 3.4 Aptamer-like functions of tsRNAs

Beyond their canonical roles in gene silencing, tsRNAs—particularly longer fragments such as tRNA halves that cannot be loaded into AGO proteins—can function through aptamer-like mechanisms dependent on their secondary and tertiary structures rather than sequence complementarity [57]. These tsRNAs fold into specific three-dimensional conformations that enable direct binding to diverse protein targets, modulating cellular processes independently of the RNAi machinery [58].

A growing body of evidence has identified tsRNAs as endogenous ligands for Toll-like receptors (TLRs), particularly TLR7 and TLR8. The tRNAVal half functions as a potent endogenous TLR7 ligand, characterized by a 5′-terminal universal sequence signature [59]. Similarly, 5′tsRNA-HisGUG is significantly upregulated upon lipopolysaccharide stimulation and potently activates TLR7 (but not TLR8) through its GU-rich sequence [60]. Notably, full-length tRNA-HisGUG fails to stimulate TLR7, indicating that this immunostimulatory activity is contingent upon the specific three-dimensional structure adopted by the tsRNA fragment rather than the linear sequence alone. Given that TLR7/8-mediated inflammatory signaling has been implicated in retinal diseases such as AMD and DR, tsRNAs released under conditions of hypoxia, oxidative stress, or inflammation may act as endogenous danger signals, activating TLR7/8 in retinal microglia, macrophages, or endothelial cells. This would amplify local inflammatory responses and synergize with VEGF-driven angiogenic signals, thereby exacerbating disease progression.

### 3.5 Stress granules

Stress granules are cytoplasmic ribonucleoprotein complexes with cytoprotective and pro-survival properties. Under conditions of oxidative stress or DNA damage, tiRNAs regulate cell survival and death by promoting the formation of stress granules. Specifically, tiRNA-5 can assemble into RNA G-quadruplex structures, which promote stress granule formation through the displacement of initiation factors and subsequent suppression of translation [61].

### 3.6 tsRNAs as carriers of epigenetic information

Beyond their somatic regulatory roles, tsRNAs have emerged as crucial carriers of epigenetic information mediating intergenerational inheritance. Sperm tsRNAs can encode and transmit paternal environmental experiences—including diet, stress, and aging—to offspring, shaping metabolic and behavioral phenotypes [62,63]. This inheritance depends critically on RNA modifications: Dnmt2-mediated m^5^C methylation protects tRNAs from fragmentation, and its deficiency abolishes the transmission of metabolic phenotypes [64].

This paradigm extends beyond metabolism to neuropsychiatric traits. Paternal stress alters sperm tsRNA profiles, and injection of these tsRNAs recapitulates anxiety-like behaviors in offspring [65,66]. Advanced paternal age similarly alters sperm tsRNA profiles, and age-mimicking tsRNA cocktails induce transcriptomic changes in embryonic stem cells mirroring pathways dysregulated in offspring of aged fathers [38]. In summary, tsRNAs function as molecular vectors of paternal epigenetic memory through combined changes in expression and modification status, establishing a "sperm RNA code" that programs offspring health.

## 4 Pathological neovascularization in ocular diseases

Neovascularization is a highly complex process that relies on precise coordination among various vascular cells, the extracellular matrix, and growth factors [67]. Under pathological conditions such as hypoxia or inflammation, local cells may upregulate the expression of angiogenic factors. This shift disrupts the equilibrium between pro- and anti-angiogenic mediators, leading to the aberrant development of new blood vessels [68]. This process primarily depends on the differentiation of endothelial progenitor cells (EPCs) and can be divided into four key steps: First, factors like VEGF and TGF-β activate matrix metalloproteinases to degrade basement membrane components (e.g., collagen and fibronectin). Next, endothelial cells migrate toward the injured area guided by chemokines. Subsequently, PDGF and other factors promote endothelial cell proliferation and initial vascular structure formation. Finally, pericytes gradually detach, while molecules such as Ang1 facilitate vessel maturation and stabilization via the Tie2 receptor signaling pathway, ultimately completing the construction of functional lumens [69].

Pathological neovascularization is closely associated with multiple blinding eye diseases [70]. Retinal vascular diseases often manifest as vascular leakage and/or abnormal neovascularization (NV), while choroidal vascular diseases are characterized by the emergence of new vessels in originally avascular subretinal spaces. Although the pathogenesis of these diseases varies, they share pathological vascular proliferation as a common core feature, which can eventually lead to severe vision loss or even blindness [71].

### 4.1 Key molecular pathways in pathological neovascularization

#### 4.1.1 VEGF-VEGFR system

The VEGFR signaling pathway plays a central role in the interplay between angiogenesis and inflammatory responses. This pathway not only promotes the migration, activation, and release of inflammatory mediators by mononuclear phagocytes but also drives neovascularization through mechanisms such as enhancing vascular permeability, degrading the extracellular matrix, and facilitating endothelial cell migration and proliferation [72]. VEGF receptors belong to the tyrosine kinase family and are transmembrane receptors critical for both angiogenesis and lymphangiogenesis. Their activity is regulated by VEGF mitogens (signaling molecules such as VEGF A–D), which contribute critically to vascular homeostasis, development, and multiple disease processes [73]. VEGF-A can bind to both VEGFR1 and VEGFR2, participating in retinal development, homeostasis, and disease. Among these, VEGFR2 is the most extensively studied receptor; it possesses strong tyrosine kinase activity and serves as a core regulator of VEGF-induced angiogenesis. In DR, the VEGFR2-mediated signaling pathway significantly promotes pathological angiogenesis and increased vascular permeability, leading to excessive neovascularization [74].

Numerous preclinical research has established the central importance of VEGF in ocular neovascularization, driving the clinical translation of anti-VEGF therapies. For conditions such as DR, retinal vein occlusion (RVO), AMD, and retinopathy of prematurity (ROP), combination treatment strategies are increasingly becoming an important approach to enhance the efficacy of monotherapies [75].

#### 4.1.2 Ang/Tie signaling pathway

The ANG/Tie system regulates endothelial cell development and angiogenesis, participates in structural remodeling from capillaries to veins under inflammatory conditions, and maintains vascular integrity [76]. Among its components, the ANG-Tie2 pathway is a well-characterized mechanism critical for vascular stability and endothelial quiescence [77]. Ang1 functions as a Tie2 agonist, triggering receptor phosphorylation and subsequent activation of the PI3K/Akt cascade. This signaling enhancement supports endothelial cell viability, stabilizes intercellular junction complexes, and suppresses migratory activity. Studies have shown that Ang1 can antagonize VEGF-mediated angiogenic activity in various disease models [78]. In contrast, Ang2 acts as an antagonist of the Tie2 receptor in quiescent endothelial cells. Its binding to Tie2 disrupts Ang1-mediated Tie2 clustering, blocks vascular stabilization, and ultimately leads to abnormal vascular structures [79].

Substantial evidence indicates that alterations in the angiopoietin signaling pathway play a key role in diabetic vascular complications. Advanced glycation end products can upregulate Ang2 expression in endothelial cells, leading to apoptosis and endothelial dysfunction. Elevated Ang2 levels also impair VE-cadherin function, resulting in increased vascular permeability [80]. Furthermore, Ang2 promotes loss of vascular wall stability and disrupts endothelial-pericyte interactions, thereby enhancing VEGF-induced neovascularization [81]. Research demonstrates that local delivery of Ang2-specific antibodies can effectively suppress pathological angiogenesis [82]. Emerging evidence indicates that concurrently targeting both Ang2 and VEGF-A could lead to superior efficacy compared to anti-VEGF monotherapy in the treatment of neovascular age-related macular degeneration [83].

#### 4.1.3 HIF-1 signaling pathway

HIF-1 is a heterodimeric transcription factor consisting of an oxygen-labile subunit (HIF-1α) and a constitutively nuclear-localized subunit (HIF-1β). The expression of HIF-1β remains unaffected by oxygen concentration and, upon binding with HIF-1α, forms a transcriptionally active complex [84]. Under normoxic conditions, the HIF-1α protein undergoes rapid degradation via the ubiquitin-proteasome pathway. In contrast, under hypoxic conditions, HIF-1α escapes degradation, translocates into the nucleus, and dimerizes with HIF-1β. This dimer specifically recognizes hypoxia response elements (HREs) in the promoter regions of target genes, initiating the transcription of downstream genes that regulate various biological processes, including apoptosis, proliferation/survival, and angiogenesis [85]. In retinal ischemic conditions, the hypoxic microenvironment facilitates both HIF-1α accumulation and the upregulation of numerous vasoactive mediators. These changes collectively induce vascular permeability and stimulate the emergence of new blood vessels [86]. Furthermore, HIF-1α exacerbates retinal damage by enhancing vascular permeability, promoting inflammatory responses, and contributing to exudative pathologies [87].

#### 4.1.4 Other signaling pathways

The Notch signaling pathway exerts a dual regulatory influence in OND, participating in the precise modulation of angiogenesis while also being implicated in inflammatory and other disease-related mechanisms [88]. During angiogenesis, Notch ligands Dll4 and Jagged1 play critical roles. When VEGF activates endothelial cells, cells with high Dll4 expression inhibit the angiogenic activity of neighboring cells through the Notch signaling pathway, thereby preventing disordered vascular growth [89]. Dll4/Notch1 knockout mice exhibit excessive retinal vascular branching and vascular leakage [90].

The PDGF family comprises four homodimeric ligands (PDGF-AA, -BB, -CC, -DD) and one heterodimer (PDGF-AB). PDGF-B has recently been recognized as essential for the integrity of the retinal vasculature. Its upregulation, along with that of its receptors, is commonly detected across multiple proliferative retinal diseases [89]. Research further demonstrates that PDGF-B enhances the proliferative, migratory, and tubulogenic abilities of retinal microvascular endothelial cells (RMECs)—effects that are suppressed by PDGFR inhibition [91].

## 5 tsRNAs and ocular neovascular diseases

### 5.1 Search strategy

**Fig. 2:**
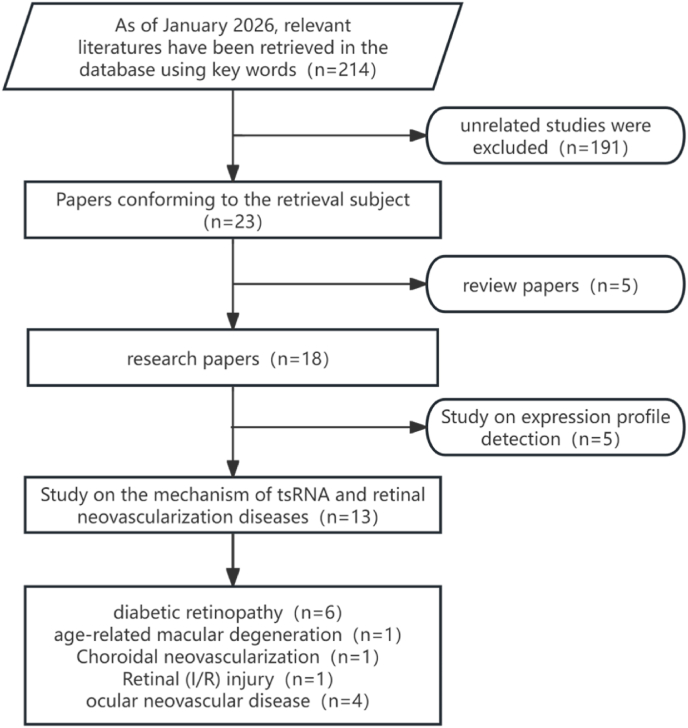
Literature Search Strategy on tsRNAs and Ocular Neovascular Diseases.

A systematic literature search was performed in PubMed and Web of Science up to January 2026. The search strategy was as follows:(((transfer RNA-derived small RNA)OR(tRNA-derived stress-induced RNA)OR(tRNA)OR(tRNA-derived fragment)OR(tRF)OR(tsRNA))AND((Retinal neovascular disease)OR(Diabetic retinopathy)OR(age-related macular degeneration)OR(Neovascular Glaucoma)OR(Retinopathy of prematurity)OR(Choroidal neovascularization)OR(Retinal ischemic pathologies)OR(Corneal Neovascularization)))”. A total of 214 articles were retrieved (PubMed: n = 148; Web of Science: n = 98), with 32 duplicates removed. Among these, 23 studies specifically investigated the relationship between tsRNAs and ONDs. Of these, 5 review articles discussed significantly altered tsRNAs in diabetes and its complications; 5 studies identified differential tsRNA expression profiles in OND-related samples, and the remaining 13 papers described specific tsRNA alterations and their underlying mechanisms. The detailed screening process is summarized in Fig. 2.

### 5.2 Expression patterns of tsRNAs in ONDs

To elucidate the role of tsRNAs in retinal neovascularization (RNV), researchers performed small RNA sequencing in an oxygen-induced retinopathy (OIR) mouse model and identified 45 significantly differentially expressed tsRNAs. These tsRNAs were predicted to target genes involved in developmental processes, suggesting a potential role in regulating retinal neovascular development. Further studies indicated that tsRNAs may participate in the molecular mechanisms underlying RNV by binding to proteins and modulating their interactions [92]. Choroidal neovascularization (CNV) represents a key pathological manifestation in NV-AMD. In the retinal pigment epithelium-choroid-sclera complex of CNV mouse models, 72 significantly altered tsRNAs were identified. GO enrichment analysis indicated that the identified target genes were predominantly linked to immune system regulation, extracellular localization, and core promoter binding functions. Meanwhile, KEGG pathway analysis indicated significant enrichment in hematopoietic cell lineage and NOD-like receptor signaling pathways [93].

Recent clinical studies have further confirmed altered tsRNA expression profiles in human retinal vascular diseases. In patients with DR, significantly dysregulated tsRNAs were detected in both vitreous humor and peripheral blood mononuclear cells (PBMCs). Their target genes were mainly enriched in cellular macromolecule metabolic processes, cytoplasmic localization, ion binding, and were significantly associated with the AMPK signaling pathway and Th17 cell differentiation [94,95]. Similarly, in PBMCs from patients with retinopathy of prematurity, six aberrantly expressed tsRNAs were identified, with target genes enriched in cellular macromolecule metabolism, intracellular anatomical structures, nucleic acid binding in transcriptional regulatory regions, and Th17 cell differentiation [96]. In summary, tsRNAs are widely dysregulated across multiple retinal neovascular diseases and contribute to pathogenic mechanisms by modulating immune responses, metabolic processes, developmental programs, and inflammatory pathways such as Th17 differentiation and AMPK signaling. These findings highlight the potential of tsRNAs as biomarkers and therapeutic targets in ocular neovascular disorders.

### 5.3 Clinical relevance of tsRNAs in ONDs

**Fig. 3:**
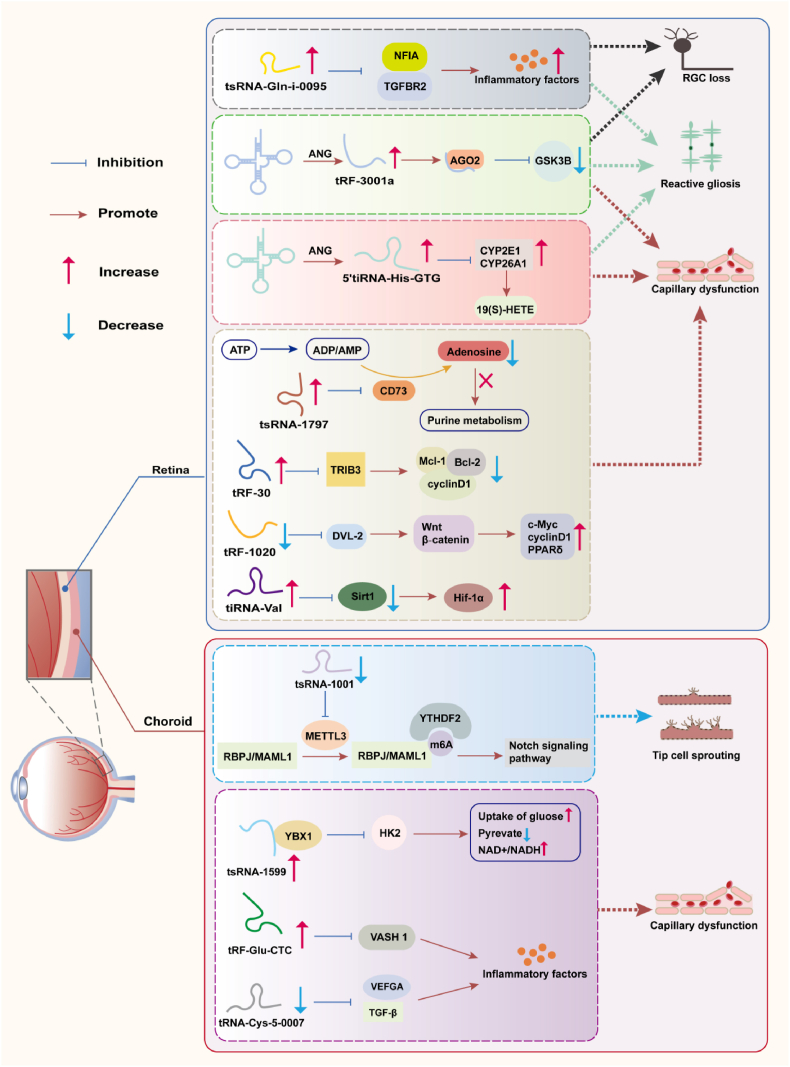
Schematic Illustration: Mechanisms of tsRNA in Ocular Neovascularization.

The association between tsRNAs and ONDs is summarized in Table 1, which integrates findings from 11 studies. The key mechanisms of tsRNAs in ONDs are summarized in Fig. 3.

**Table 1:** The mechanisms and effects of tsRNAs in ONDs.

Disease	tRFs	Trends	Upstream	Target genes	Downstream	Pathway	Effects or Biological Phenotypes
DR	tRF-3001a [97]	UP	ANG	GSK3B	-	-	Retinal glial activation and endothelial angiogenic effects, thereby causing retinal neurodegeneration, vascular leakage, and acellular capillary generation
DR	tsRNA-1797 [98]	UP	-	CD 73	Adenosine	Purine metabolism	Reduced adenosine production and causing retinal vascular dysfunction in DR.
DR	5′tiRNA-His-GTG [99]	UP	-	CYP2E1	19(S)-HETE	Arachidonic acid metabolism	Accelerates retinal vascular dysfunction, exacerbates retinal neurodegeneration, and impairs visual function and visually-guided behaviors
				CYP26A1			
DR	tRF-30 [100]	UP	-	TRIB3	Mcl-1, cyclin D1, and Bcl-2	STAT3	Induced retinal vascular complications
DR	tRF-1020 [101]	Down	-	DVL‐2	Wnt/β‐catenin	c-Myc,cyclinD1, PPARδ	Alleviated retinal vascular dysfunction as shown by decreased retinal acellular capillaries, vascular leakage, and inflammation
DR	tiRNA-Val [102]	UP	ANG	Sirt 1	Hif-1α	Hif-1α	Proliferation of human retinal endothelial cells
AMD	tRF-Glu-CTC [103]	UP	ANG	VASH 1	-	-	Released inflammatory factors and induced angiogenic phenotypes in endothelial cells
AMD	tRF-1001 [104]	Down	METTL3	RBPJ	-	Notch signaling pathway	Decreased vasopermeability and reduced the number of neovascular tufts
				MAML1			
CNV	tsRNA-1599 [13]	UP	YBX1	HK2	-	-	Promoted angiogenic effects in endothelial cells in vitro and enhanced pathological ocular angiogenesis in vivo
OND	tRNA-Cys-5-0007 [105]	Down	-	VEGFA	-	-	Mitigated pathological angiogenesis, vascular leakage, and inflammatory responses
				TGF-β1			
Retinal (I/R) injury	tsRNA-Gln-i-0095 [106]	UP	-	NFIA	-	-	Drives retinal glial activation, inflammatory responses, and subsequent retinal neurodegeneration
				TGFBR2			

AMD, age-related macular degeneration; DR, diabetic retinopathy; CNV, choroidal neovascularization; OND, ocular neovascular disease; Retinal (I/R) injury: Retinal ischemia-reperfusion injury.

#### 5.3.1 tsRNAs and retinal neovascularization

During the preclinical stage of DR, reduced retinal blood flow, inner retinal neuronal dysfunction, and diminished glial/microglial reactivity can already be observed, indicating impaired neurovascular unit function early in the disease course [107]. Multiple studies have demonstrated that tsRNAs play important regulatory roles in the pathogenesis and progression of DR. For instance, tRF-3001a contributes to vascular dysfunction by disrupting the retinal neurovascular unit. Conversely, its inhibition alleviates reactive astrogliosis, protects retinal ganglion cells, and maintains visual function [97]. Metabolic dysregulation is a key driver of DR pathogenesis. Plasma metabolomic profiling in diabetic patients has revealed numerous metabolites closely associated with vascular complications [108]. tsRNA-1797 impairs purine metabolism in vascular endothelial cells through direct interaction with CD73, resulting in diminished adenosine generation and consequent retinal vascular impairment [98]. 5′tiRNA-His-GTG acts as a key modulator of neurovascular dysfunction, directly modulating Müller cell function and indirectly influencing angiogenic responses and retinal ganglion cell survival. Its mechanism primarily involves the regulation of arachidonic acid metabolism via the CYPs pathway, thereby mitigating retinal vascular and neuronal injury and promoting visual recovery [99]. Inflammation, as a core pathogenic mechanism in DR, persists throughout the disease course and upregulates various inflammatory mediators including chemokines, cytokines, transcription factors, growth factors, and VCAM-1 [109]. tRF-30 exacerbates the retinal inflammatory microenvironment and promotes pathological neovascularization through activation of the STAT3 signaling pathway [100]. tsRNAs also contribute to neovascular regulation by disrupting the balance between pro-angiogenic and anti-angiogenic factors. For example, tRF-1020 alleviates retinal vascular leakage, inflammation, and aberrant angiogenesis in diabetic mice, and inhibits high glucose-induced dysfunction in human retinal microvascular endothelial cells (HRMECs). This occurs through direct targeting and suppression of VEGFA expression, thereby inhibiting hyperactivation of the VEGF signaling pathway [101]. Furthermore, Hif-1α, a key mediator in retinal neovascularization and DR, is indirectly stabilized by tiRNA-Val through the inhibition of Sirt1, resulting in the activation of downstream pro-angiogenic signaling pathways [102,110].

#### 5.3.2 tsRNAs and choroidal neovascularization

NV-AMD accounts for approximately 15% of all AMD cases and represents a major subtype leading to severe vision loss in the elderly population [111]. Endothelial cell dysfunction and pathological vascular remodeling represent characteristic pathological changes in age-related ocular disorders, highlighting these cells as a potential therapeutic focus for related conditions [112]. Vasohibin (VASH), including VASH-1 and VASH-2, was initially identified as a regulator of angiogenesis, and its dysregulation is closely associated with aberrant angiogenic processes [113]. Research has shown that tRF-Glu-CTC influences endothelial cell activity by enhancing the release of pro-angiogenic mediators and downregulating VASH1 expression, which facilitates neovascularization. Suppressing tRF-Glu-CTC activity retards choroidal neovascularization (CNV) progression and alleviates retinal injury caused by prolonged hypoxic stress [103]. tRF-1001 exerts anti-angiogenic effects during ocular vascular development. It facilitates m^6^A modification of mRNAs encoding RBPJ and MAML1—key transcriptional regulators in the Notch signaling pathway—through a METTL3-dependent mechanism, and recruits YTHDF2 to suppress their expression, ultimately inhibiting sprouting of choroidal vascular endothelial cells and pathological angiogenesis [104]. tsRNA-1599 promotes pathological angiogenesis by interacting with the transcription factor YBX1, thereby altering the expression of critical glycolytic enzymes HK2 and PKM2 in endothelial cells and modulating glycolytic flux [13]. Meanwhile, tRNA-Cys-5-0007 exhibits dual anti-angiogenic functions: on one hand, it inhibits endothelial cell proliferation, migration, tube formation, and sprouting by downregulating VEGFA expression; on the other hand, it attenuates ocular vascular leakage, pathological angiogenesis, and leukocyte infiltration in the retina and choroid via suppression of TGF-β1 expression [105].

#### 5.3.3 tsRNAs and ischemia-reperfusion injury

The core pathophysiological mechanism underlying glaucoma, a progressive and irreversible optic neuropathy, centers on retinal ischemia-reperfusion (I/R) injury. The disease often results from a sharp increase in intraocular pressure, which compresses retinal capillaries and leads to acute retinal ischemia and hypoxia. Consequently, acute retinal ischemia and hypoxia occur, ultimately resulting in the degeneration of retinal ganglion cells (RGCs) and damage to the optic nerve [114]. Similar I/R injury mechanisms are also widely observed in other ocular diseases, such as retinal artery occlusion, central retinal vein occlusion, and retinopathy of prematurity. The progression of retinal I/R injury encompasses a range of pathological mechanisms, including oxidative stress, immune dysregulation, disruption of the inner blood-retinal barrier (iBRB), neurovascular unit (NVU) dysfunction, and reactive gliosis [115]. Recent studies indicate that tsRNA-Gln-i-0095 plays a critical role in regulating glial cell activation and inflammatory responses during retinal I/R injury. Downregulation of tsRNA-Gln-i-0095 expression significantly suppresses reactive gliosis in retinal Müller cells, reduces the release of inflammatory factors, and thereby effectively preserves the structure and function of RGCs [106].

## 6 Clinical application prospects of tsRNAs

### 6.1 Delivery strategies

#### 6.1.1 Viral vectors

The eye represents a unique target organ at the forefront of gene therapy development. Viral vectors, particularly adeno-associated viruses (AAVs), have emerged as the platform of choice for ocular gene therapy due to their favorable safety profile and capacity for long-term, stable gene expression [116]. AAVs efficiently transduce non-dividing target cells, such as retinal pigment epithelium (RPE) cells and photoreceptors, a feature particularly crucial for chronic ocular neovascular diseases (ONDs) that require sustained modulation of pathological angiogenesis. The primary advantage of lentiviral vectors lies in their packaging capacity. Despite their larger cargo capacity, carry inherent risks of insertional mutagenesis, warranting more stringent safety considerations in ophthalmic applications [117]. Adenoviral vectors, owing to their substantial immunogenicity, are less suitable for chronic conditions requiring long-term, stable therapeutic intervention.

From the perspective of tsRNA delivery, viral vectors offer considerable potential. First, tsRNA molecules are inherently short (typically 14−40 nt), well below the ∼4.7 kb packaging limit of AAV, rendering the delivery of tsRNA precursor sequences via AAV technically feasible. By selecting AAV serotypes with specific tropism, precise tsRNA expression at pathological sites can be achieved, thereby minimizing off-target effects on surrounding healthy tissues. However, the application of viral vectors for tsRNA-based therapy also presents unique challenges. The biogenesis of tsRNAs involves specific enzymatic cleavage, necessitating that tsRNA precursors delivered via viral vectors be correctly recognized and processed within target cells to generate functional mature fragments. More importantly, as multi-target regulatory molecules, long-term overexpression of tsRNAs may perturb physiological functions, particularly under pathological conditions such as oxidative stress or inflammation. These potential long-term safety concerns warrant thorough evaluation in future preclinical studies.

#### 6.1.2 Non-viral vectors

Non-viral vectors offer scalable, low-immunogenicity platforms with excellent production reproducibility, providing an important technological alternative for ocular gene therapy. Although their delivery efficiency was historically limited compared to viral vectors, substantial progress has been made in recent years. Lipid-based nanoparticles (LNPs) and liposomes currently represent the most advanced non-viral delivery systems, capable of efficiently transfecting retinal cells and achieving long-term gene expression [118]. Recent studies have demonstrated that LNPs can penetrate the neural retina and successfully deliver mRNA to photoreceptor cells, opening new avenues for treating inherited blinding disorders [119]. For instance, ECO/pRHO-ABCA4 nanoparticles achieved sustained ABCA4 expression and significantly delayed disease progression in a mouse model of Stargardt disease [120]. Polymer-based vectors, such as polyethyleneimine (PEI), have been optimized through biocompatibility modifications to enhance delivery efficiency while reducing toxicity, showing promising application prospects. These vectors can encapsulate and deliver genetic material to target cells, offering a scalable and flexible therapeutic approach, with preclinical studies confirming long-term expression of therapeutic genes in the retina [121]. Although non-viral vectors currently face challenges of lower transduction efficiency compared to viral vectors, ongoing research efforts focused on enhancing vector stability, improving targeting specificity, and reducing potential toxicity are driving these systems toward clinical translation, ultimately aiming to achieve safe and effective applications in posterior segment gene therapy.

#### 6.1.3 Exosomes

As natural intercellular communication vehicles, exosomes exhibit unique potential for tsRNA delivery. Compared to synthetic LNPs, exosomes possess inherent targeting capabilities and low immunogenicity; relative to viral vectors, they offer greater cargo flexibility and absence of genomic integration risks [1]. Studies have confirmed that the use of exosome formulations further enhances the synergistic anti-angiogenic and anti-inflammatory efficacy of tsRNAs in diabetic retinopathy models [105]. This property positions engineered exosomes—loaded with specific tsRNA mimics or inhibitors—as a novel class of ocular targeted delivery platforms that mimic natural intercellular communication, enabling precise regulation of pathological angiogenesis.

## 7 Conclusion

Current clinical management of ONDs primarily relies on laser photocoagulation, intravitreal anti-VEGF injections, and vitrectomy [122]. Despite their widespread use, long-term anti-VEGF therapy is associated with several limitations, including suboptimal patient response, acquired drug resistance, and adverse effects such as retinal atrophy [123]. In this context, tsRNAs have emerged as promising regulators of pathological angiogenesis, offering the potential for multi-target synergistic modulation. Notably, tsRNA-mediated post-transcriptional regulation may provide greater efficiency and flexibility in coordinating the expression of multiple genes compared to transcriptional-level interventions [124,125].

tsRNAs play essential roles in diverse biological processes—including early embryonic development, stem cell fate determination, immune responses, and viral infections—by modulating gene expression at transcriptional, post-transcriptional, and translational levels. Recent advances have significantly expanded our understanding of tsRNA biogenesis and function in ONDs. However, compared to well-characterized non-coding RNAs such as miRNAs and piRNAs, research on tsRNAs remains in its early stages, and many fundamental questions regarding their regulatory mechanisms remain unresolved.

Several limitations in current tsRNA research must be acknowledged. Most studies are based on small-sample clinical observations or animal models, lacking validation in large-scale, multicenter cohorts. In addition, the potential interplay between tsRNAs and other non-coding RNAs—such as microRNAs and long non-coding RNAs—remains largely unexplored. Elucidating these complex regulatory networks will be essential for a comprehensive understanding of the molecular architecture underlying ONDs.

Future research should prioritize the design and implementation of multicenter clinical trials to evaluate the diagnostic and prognostic value of tsRNAs as biomarkers. Advances in targeted delivery systems, including nanoparticle-based and viral vector-mediated approaches, will be critical for translating tsRNA-based therapies into clinical practice. Furthermore, integrative analyses combining tsRNA, mRNA, and protein interaction networks will help uncover broader regulatory landscapes and facilitate the development of combination strategies targeting multiple pathogenic pathways simultaneously. Addressing these challenges will pave the way for tsRNAs to emerge as a novel class of therapeutic agents in the management of ONDs.

## CRediT authorship contribution statement

**Tian-yi Liu:** Writing – original draft, Visualization, Supervision, Software, Investigation, Formal analysis, Data curation, Conceptualization. **Peng-zhou Kuai:** Visualization, Investigation, Data curation. **Yi-sheng Luo:** Visualization, Investigation, Data curation. **Yu Song:** Writing – review & editing, Conceptualization. **Yong Wang:** Writing – review & editing, Supervision, Conceptualization. **Xiao-bo Huang:** Writing – review & editing, Methodology, Conceptualization. **Xin Cao:** Writing – review & editing, Writing – original draft, Supervision, Funding acquisition, Conceptualization.

## Availability of data and materials

The data that support the findings of this study are available from the corresponding author upon reasonable request.

## Funding

This work was supported by 10.13039/501100001809National Natural Science Foundation of China (82501314); Jiangsu Provincial Health Commission Scientific Research Project (M2024093); Natural Science Foundation of Science and Technology Bureau of Nantong City (JC2023028).

## Declaration of competing interest

The authors declare that they have no known competing financial interests or personal relationships that could have appeared to influence the work reported in this paper.

## Abbreviation

(OND): Ocular neovascular diseases
(VEGF): Vascular endothelial growth factor
(tsRNAs): Transfer RNA-derived small RNAs
(DR): Diabetic retinopathy
(NV-AMD): Neovascular age-related macular degeneration
(CoNV): Corneal neovascularization
(ANG): Angiopoietin
(PDGF): Platelet-derived growth factor
(sncRNAs): Small non-coding RNAs
(tRFs): tRNA-derived fragments
(i-tRFs): Internal tRFs
(tiRNAs): tRNA-derived stress-induced RNAs
(m^5^U): 5-Methyluridine
(AGO): Argonaute
(RISC): RNA-induced silencing complex
(3′ UTRs): 3′ untranslated regions
(RBPs): RNA-binding proteins
(YBX1): Y-box binding protein 1
(EPCs): Endothelial progenitor cells
(RVO): Retinal vein occlusion
(ROP): Retinopathy of prematurity
(HREs): Hypoxia response elements
(RNV): Retinal neovascularization
(OIR): Oxygen-induced retinopathy
(CNV): Choroidal neovascularization
(I/R): Ischemia-reperfusion
(RGCs): Retinal ganglion cells
(iBRB): inner blood-retinal barrier
(NVU): Neurovascular unit
